# The antiviral activity of a small molecule drug targeting the NSP1-ribosome complex against Omicron, especially in elderly patients

**DOI:** 10.3389/fcimb.2023.1141274

**Published:** 2023-03-07

**Authors:** Min Shen, Ping Ding, Guangxin Luan, Ting Du, Shanshan Deng

**Affiliations:** ^1^ Non-coding RNA and Drug Discovery Key Laboratory of Sichuan Province, Chengdu Medical College, Chengdu, Sichuan, China; ^2^ Vincent Mary School of Science and Technology, Assumption University, Bangkok, Thailand

**Keywords:** COVID-19, Nsp1, translational inhibition, elderly patients, Omicron infection, XBB sub-variant

## Abstract

**Introduction:**

With the emergence of SARS-CoV-2 mutant strains, especially the epidemic of Omicron, it continues to evolve to strengthen immune evasion. Omicron BQ. 1 and XBB pose a serious threat to the current COVID-19 vaccine (including bivalent mRNA vaccine for mutant strains) and COVID-19-positive survivors, and all current therapeutic monoclonal antibodies are ineffective against them. Older people, those with multimorbidity, and those with specific underlying health conditions remain at increased risk of COVID-19 hospitalization and death after the initial vaccine booster. However, small-molecule drugs for conserved targets remain effective and urgently needed.

**Methods:**

The non-structural protein of SARS-CoV-2 non-structural protein 1(Nsp1) can bind to the host 40S ribosomal subunit and activate the nuclease to hydrolyze the host RNA, while the viral RNA is unaffected, thus hijacking the host system. First, the present study analyzed mutations in the Nsp1 protein and then constructed a maximum-likelihood phylogenetic tree. A virtual drug screening method based on the Nsp1 structure (Protein Data Bank ID: 7K5I) was constructed, 7495 compounds from three databases were collected for molecular docking and virtual screening, and the binding free energy was calculated by the MM/GBSA method.

**Results:**

Our study shows that Nsp1 is relatively conserved and can be used as a comparatively fixed drug target and that therapies against Nsp1 will target all of these variants. Golvatinib, Gliquidone, and Dihydroergotamine were superior to other compounds in the crystal structure of binding conformation and free energy. All effectively interfered with Nsp1 binding to 40S protein, confirming the potential inhibitory effect of these three compounds on SARS-CoV-2.

**Discussion:**

In particular, Golwatinib provides a candidate for treatment and prophylaxis in elderly patients with Omicjon, suggesting further evaluation of the anti-SARS-CoV-2 activity of these compounds in cell culture. Further studies are needed to determine the utility of this finding through prospective clinical trials and identify other meaningful drug combinations.

## Introduction

Since November 9, 2021, a new novel coronavirus variant has been detected in South Africa, and more than 300 variants of subtype Omicron have appeared. Novel coronavirus variant has caused several outbreaks worldwide and continued to evolve toward strengthening immune evasion. Major epidemic strains in many countries worldwide have been replaced by BQ. 1 and XBB families. XBB mutant strain was first discovered in India in August 2022. It spread rapidly and soon became a major epidemic strain in Singapore and other countries. Novel Coronavirus Omicron new subtype BQ. 1.1 is resistant to all currently known monoclonal antibody therapies (Cao et al., 2022; Roohani and Keikha, 2022; Arora et al., 2023). According to the Global Initiative for Shared Influenza Data (GISAID), XBB. 1.5 has been identified in at least 74 countries and territories since December 31, 2022. Symptoms of infection by the XBB subtype strain include dyspnea, headache, sore throat, stuffy congestion, general pain, fatigue, and fever. The increase in mutant strains such as the Omicron subtype strain XBB. 1.5 may further undermine the efficacy of the current COVID-19 vaccine and lead to a surge in breakthrough infections and re-infections. At the same time, on October 14, 2022, studies suggested that age over 80, suffering from a variety of underlying diseases, the use of immunosuppressants, chronic kidney disease, and other factors are the risk factors for developing severe disease after novel coronavirus infection, so these people need additional vaccination (Agrawal et al., 2022; Bao et al., 2022).

However, the Omicron BA.5 subclade BA.5.2 and BF.7 are still the most prevalent in China, indicating that herd immunity is weak for new mutants that evade the immune barrier established by prior vaccination and natural infection, and mRNA vaccines, similar to inactivated vaccines, have a limited role in preventing XBB and BQ. 1.1 breakthrough infection. The rise of these new variants is mainly attributed to mutations in the receptor binding domain (RBD) of the viral spike protein, which helps novel coronavirus evade the COVID-19 vaccine and neutralizing antibodies induced by natural infection. The more RBD mutations, the faster the mutant strain seems to rise. BQ. 1.1 has six RBD mutations, one more critical mutation than BQ. 1, while the RBD mutations in XBB have seven. These new RBD mutations in the Omicron mutant strains have a convergence effect. Overall, RBD has 7–10 identical mutation sites, which were not present before the COVID-19 Outbreak, demonstrating that the convergent evolution mutation sites have a high selection advantage and that there is a great deal of concentrated evolutionary pressure, which will eventually result in convergence evolution and evolution (Davis-Gardner et al., 2022; Zheng et al., 2022). Because these strains have many mutations, they could lead to less effectiveness of the COVID-19 vaccine and more breakthrough infections (Kurhade et al., 2022).

Vaccination has been widely promoted as an important preventive measure against COVID-19. Among clinically developed vaccines, protein subunit (32%), RNA (23%), viral vector (non-replication) (13%), inactivated virus (13%), DNA (9%), and other types of vaccines. As research on protein subunit vaccines was relatively mature and was the priority vaccine development method, the number of protein subunit vaccines was the largest among COVID-19 vaccines. However, persistent mutations of the virus can affect the vaccine’s preventive effect, especially Omicron, which largely evaded the antibodies elicited by the vaccine (Planas et al., 2021).

SARS-CoV-2 comprises a single-stranded positive-sense RNA genome that encodes structural and non-structural proteins. Nsp1 is one of the first proteins expressed from the SARS-CoV-2 genome and is a major virulence factor for COVID-19. Studies on SARS-CoV-2 have implicated Nsp1, the first encoded viral protein, as a virulence factor with a key role in the host translation shutdown. The presence of Nsp1 in ribosomal preinitiation complexes very likely prevents the accommodation of mRNA into the entry channel of the 40S subunit to inhibit translation initiation (Lapointe et al., 2021). These previous observations support that Nsp1 plays an essential role in viral infection. Therefore, Nsp1 is currently one of the targets for new antiviral drugs. Accordingly, it is crucial to monitor Nsp1 polymorphisms to understand their role during infection and virus-induced pathogenicity.

Developing a new drug is time-consuming and requires a huge financial investment. In the current global crisis, repositioning existing drugs is a potentially useful tool for finding new treatment options. Computer-assisted virtual screening provides an inexpensive and rapid alternative to high-throughput screening for drug discovery. Furthermore, virtual screening techniques can optimize the selection of potential drugs. Over the past few decades, virtual screening has played an important role in discovering small-molecule inhibitors of therapeutic targets. Various ligands, and structure-based virtual screening methods, have been used to identify small molecule ligands for a protein of interest (Rogosnitzky et al., 2021).

Our study first analyzed the amino acid sequence mutations of the Nsp1 protein to construct a maximum-likelihood phylogenetic tree. The competitive binding of small molecules of the potential Nsp1-40S complex was investigated using docking-based virtual screening methods. We used molecular docking and molecular dynamics (MD) simulations for Nsp1 and the compound. Compounds were classified according to the combined free energy score. Potential drug compounds that could inhibit Nsp1 were identified by analyzing the binding patterns between the compounds with better scoring results and Nsp1. In parallel, we performed binding free energy calculations for complexes of eight compounds and Nsp1. This study aimed to identify potential drug compounds in the drug library by molecular docking and Nsp1 simulation, which may shed insights for future studies on small molecule medications that can be used to treat novel coronavirus infections clinically.

## Materials and methods

### Nsp1 sequence mutation analysis

Nsp1 is located in the n terminal of the ORF1ab sequence and may undergo amino acid mutation. The reference sequence is the first discovered and sequenced strain with access number “YP_009742608.1”, also known as the wild type. All of the AAS samples were compared with the reference sequence. 180 AAs of the Nsp1 region were extracted from NCBI (https://www.ncbi.nlm.nih.gov/sars-cov-2/),The Nsp1 sequences were extracted by a custom python script. The MAFFT software (ref. 1) was applied to perform a multiple sequence alignment analysis for amino acid mutations (Katoh and Standley, 2013). The AliView software (ref. 4) was used to visualize the multiple sequence alignment results (Larsson, 2014).

### Construct a maximum-likelihood phylogenetic tree

The IQTree software (ref. 2) was used to build the maximum-likelihood phylogenetic tree (Nguyen et al., 2015). The FigTree software (http://tree.bio.ed.ac.uk/software/Figtree/) was used to make the re-root tree. The R package ggtree (ref. 3) was used to visualize the tree (Yu et al., 2016).

### Protein–protein docking of 40S-SARS-CoV-2-Nsp1(BQ.1)

Homology model of the SARS-CoV-2-Nsp1(BQ.1) protein was built by modeller9.18 using the crystal structure of 40S-SARS-CoV-2-Nsp1 (PDB: 7K5I) as a template. Hundred independent structures were constructed, and the one with the best DOPE score was chosen for further energy minimization in Amber18 using an ffff14SB force field. Rosetta3.7 was used to perform protein-protein docking to get the 40S- BQ.1**-**Nsp1 complex and default values are used for parameter setting.

### Screening of small molecular ligands

Each sub-library (FDA, investigational-only, world-no-FDA) was downloaded from the zinc database (https://zinc.docking.org/). Virtual screening of the small molecule database was conducted using autodock-vina.22. Using prepare_ligand4.py scripts, small molecule mol2 files were converted to pdbqt format files and kept all rotatable keys flexible. Using the prepare_receptor4.py script, the receptor protein pdb file was converted to pdbqt files, which was used as a molecular pair acceptor. The molecular docking parameters are as follows: center_x = 4.87, center_y = -16.03, center_z = 8.314, size_x = 40, size_y = 40, size_z = 40, exhaustiveness = 32. Each small molecule will produce the affinity data. This data was sorted and filtered.

### Binding free energy calculation between 40S-SARS-CoV-2-Nsp1 (BQ.1) and small molecular ligand

The 40S-SARS-CoV-2-Nsp1 (BQ.1) crystal structure was used as a template to model the SARS-CoV-2 target 40S using modeler 9.18. One hundred independent structures were constructed, and the structures with the best DOPE score were selected to reduce the energy of Amber further. The relaxed model was saved as pdb files and converted to pdbqt format as a pair acceptor using AutoDockTools-1.5.6 with specified atomic type and partial charge. The docked minimal conformation was used as the initial position of the drug molecule. Each simulation system was immersed in a cubic cube of TIP 3 P water at a distance of 10 A from the solute. To neutralize the system with either Na^+^ or Cl^-^.The ligand and protein were parameterized with the General Amber force field (GAFF) 15 and Amber ff14SB force fields, respectively. 10,000 steps of constraint minimization (10 kcal/mol/A2), including 5000 steps of steepest descent minimization and 5000 steps of conjugate gradient minimization to optimize each system. Each system was then heated to 300k in 0.2 ns and equilibrated for 0.1 ns in the NPT ensemble. Finally, a 5ns MD simulation was performed for each system at 300k. Minimization, heating, and equilibration were performed using the sander program in Amber18. A 5-ns production run was performed using the pmemd.cuda. Based on the 5 ns MD simulation trajectory, the binding free energy (ΔG) was calculated by the MM/GBSA method with the following formula: ΔGcal=ΔEvdw+ΔEele+ΔGgb+ΔGnp-TΔS, where ΔEele and ΔEvdw refer to electrostatic and van der Waals energy terms respectively. ΔGgb and ΔGnp refer to polar and non-polar solvation-free energies, respectively.

## Results and discussion

### Comparison of amino acid sequences of Nsp1 in 23 Omicron variants

**Figure 1:**
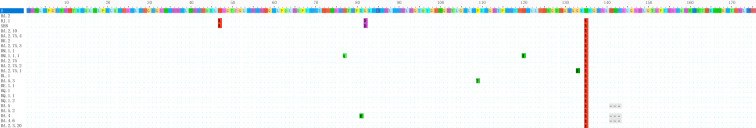
Comparison of amino acid sequences of Nsp1 in 1-180aa between 23 Omicron variants.

**Figure 2:**
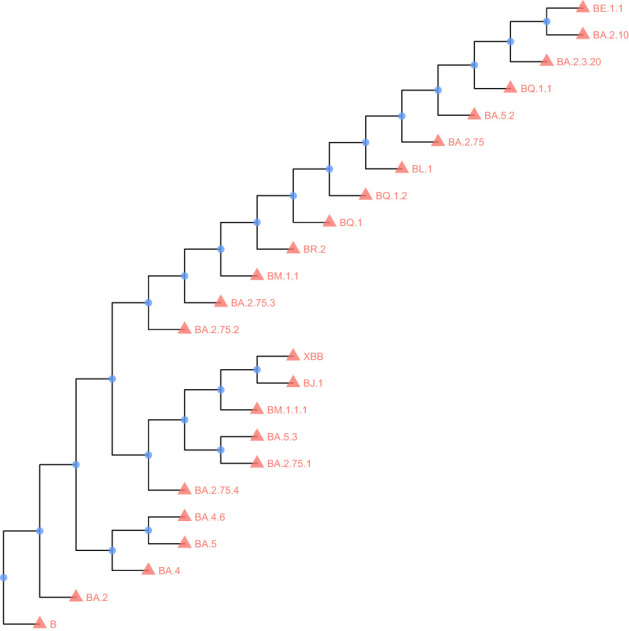
Maximum likelihood phylogenetic tree of Nsp1.

To analyze the mutation in the Nsp1 amino acid sequence, we selected 23 currently mainstream Omicron mutant strains for analysis, and the sequence of YP _ 009742608.1 was used as a control. All VOC has a common S135R mutation, and secondly, BJ. 1 and XBB have two common mutations: K47R and G82D. The strain BM.1.1.1 has amino acid mutations at two sites: R77Q and K120N. The results show that the sequence of Nsp1 is relatively conserved. The results are shown in **Figures 1**, **2**.

### Docking results of 7495 drugs against 40S-SARS-CoV-2-Nsp1 (BQ.1) model

The 7,495 drugs obtained from the zinc database were screened for molecular docking. Among them, 93 small molecule compounds have docking scores better than 7.5 kcal/mol. 24 of the 2,100 compounds approved by the FDA. There were 4,263 compounds approved by regulatory agencies other than the FDA. Among them, 25 compounds with a docking score better than -7.5 kcal/mol. 34 of the 1,132 compounds in clinical trials have a docking score better than -7.5 kcal/mol. In addition, 21 top compounds with docking scores better than -8 kcal/mol were selected from homologous model docking results (**Table 1**).

**Table 1:** Eight drugs selected from the 40S-SARS-CoV-2-Nsp1 (BQ.1) model.

No	Drug name	ID	Data	Affinity (kcal/mol)	Chemical formula
1	Cepharanthine	ZINC000030726863	World-not- FDA	-9.2	C_37_H_38_N_2_O_6_
2	Hypericin	ZINC000003780340	Investigational-only	-8.6	C_30_H_16_O_8_
3	Ergotamine	ZINC000052955754	FDA	-8.5	C_33_H_35_N_5_O_5_
4	Golvatinib	ZINC000043195317	Investigational-only	-8.5	C_33_H_37_F_2_N_7_O_4_
5	Dihydroergotoxine	ZINC000014880002	World-not- FDA	-8.2	C_34_H_41_N_5_O_8_S
6	Dihydroergotamine	ZINC000003978005	FDA	-8	C_33_H_37_N_5_O_5_
7	Zosuquidar	ZINC000100029945	Investigational-only	-8	C_32_H_31_F_2_N_3_O_2_
8	Gliquidone	ZINC000001482077	World-not- FDA	-8	C_27_H_33_N_3_O_6_S

### Docking results of eight drugs against 40S-SARS-CoV-2-Nsp1 (BQ.1) model

Paxlovid is already diagnosed with coronavirus infection treatment, which is unsuitable for prevention. The national diagnosis and treatment plan is light or common type have severe high-risk factors of adults, no high-risk factors of no need to use, need to use within 5 d, more than 5 d is affected by obvious effect, the note is mainly and many drugs conflict, need to inform the doctor at ordinary times, evaluated by the doctor whether can use Paxlovid, basic medication may need to adjust first. Therefore, medication has significant limitations (Tan et al., 2022).

**Figure 3:**
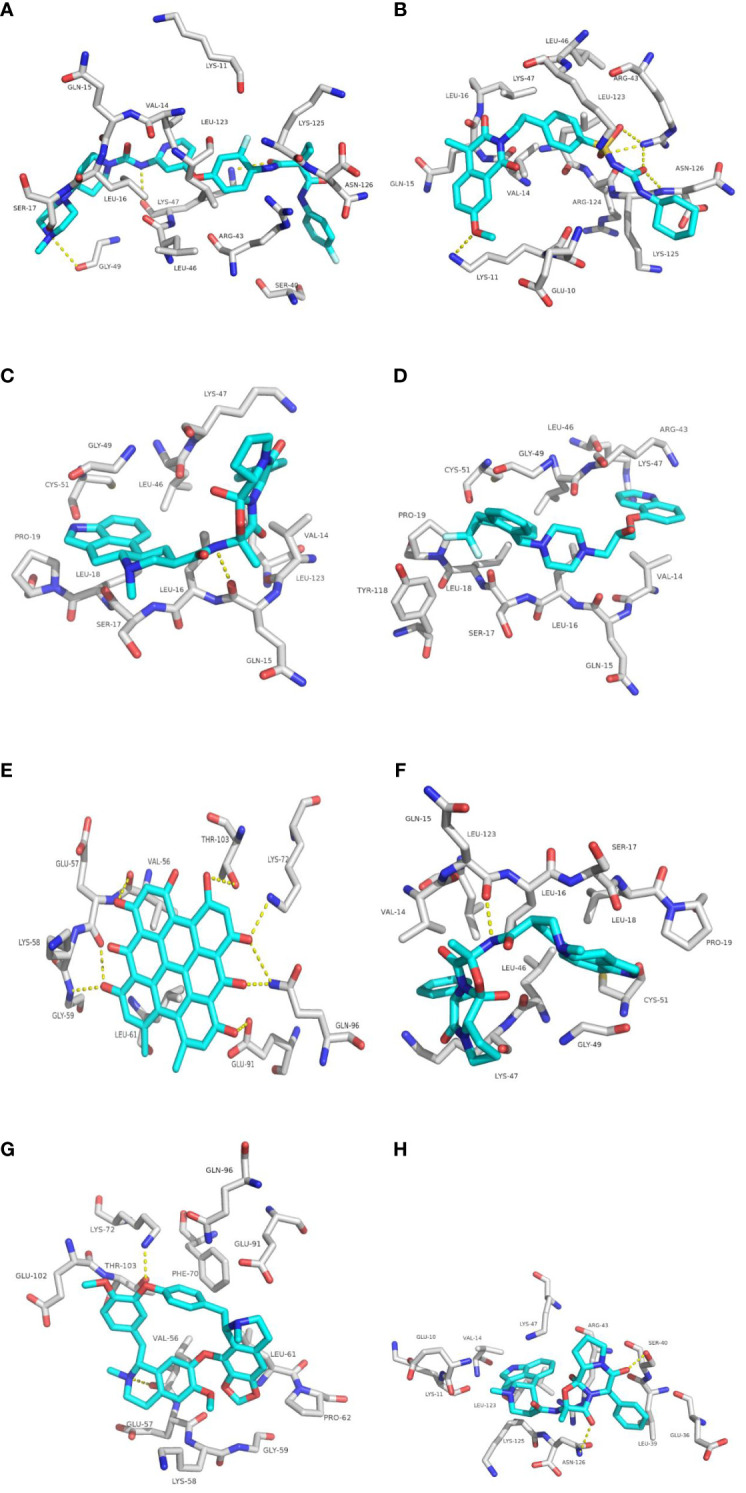
Binding model of Small molecule ligand with 40S-SARS-CoV-2-Nsp1 (BQ.1) complex interface. The yellow dotted line indicates the interaction distance (Å). **(A–H)** are Golvatinib, Gliquidone, Dihydroergotamine, Zosuquidar, Hypericin, Ergotamine, Cepharanthine, Dihydroergotoxine.

These COVID-19-infected patients need antiviral drugs or other drugs to treat SARS-CoV-2 infection. More potential compounds need to be tested. In the presence of 40S-SARS-CoV-2-Nsp1 (BQ.1) complex formation, that is, when symptoms of infection may already be present, small molecule interference with the 40S-SARS-CoV-2- Nsp1 (BQ.1) complex can be assessed by assessing the likelihood of small molecules entering the lumen of the complex. Further, eight top compounds that showed a docking score better than 8 kcal/mol were selected from the docking results of the homology model (**Table 1** and **Figure 3**). Since these drugs have previously been used in humans, much is known about their side effects. Therefore, testing these drug combinations in clinical trials may improve the possibility of COVID-19 as an effective therapeutic alternative.

Cepharanthine has been successfully applied to treat many diseases, including tuberculosis, pneumoconiosis, AIDS, and SARS (Rogosnitzky et al., 2021). Studies have shown that Cepharanthine can associate with the S protein of SARS-CoV-2 to interfere with its invasion into host cells, while nelfinavir suppresses viral replication through protease inhibition. The combination of nelfinavir oral and qianvenin intravenous administration can achieve better anti-novel coronavirus effects (Ohashi et al., 2021).

Hypericin Inhibits the protein kinase activity required for many viruses’ replication. Hypericin induces significant changes in the HIV capsid protein p24 in the presence of light and may suppress reverse transcriptase activity (Bajrai et al., 2022).

Golvatinib is a lead drug with the best effect on HAV inhibition screened through *in vitro* inhibition experiments. Related functional experiments proved that the small molecule does not cause the stability of HAV particles but also suppresses HAV infection by blocking the binding of HAV to cell surface receptors. Through *in vitro* inhibition of Golvatinib, Golvatinib (IC50 about 1uM) was selected as the lead drug with the best inhibitory effect. Relevant functional experiments showed that the small molecule did not cause changes in the stability of HAV particles but inhibited HAV infection by blocking the binding of HAV to cell surface receptors (Cao et al., 2019). A cell-based *in vitro* assay revealed that HGF/MET inhibition, induced by golvatinib, constrained the growth of chemoresistant SCLC cells through the inhibition of ERK and AKT signals. Furthermore, treatment with golvatinib suppressed the systemic metastasis of SBC-5 cell tumors in natural killer cell-depleted SCID mice, predominantly through cell cycle arrest (Nakagawa et al., 2014; Taniguchi et al., 2017).

Some studies have shown that combination of birinapant plus the MDR1 inhibitor zosuquidar is a safe and targeted therapy to kill HBV-replicating hepatocytes. Moreover, the observation that zosuquidar can safely potentiate the antiviral effect of birinapant in an *in vivo* HBV model opens a way to reduce the dose-limiting toxicities previously reported in Smac-mimetic clinical trials for HBV infection (Morrish et al., 2020).

Gliquidone, the second generation of oral sulfonylureas, a highly active pro-islet β -cell agent, combined with specific receptors on the islet β cell membrane, which can induce an appropriate amount of insulin to reduce blood glucose concentration. It has also been shown that Gliquidone can bind to the Mpro-binding pocket, suggesting that it may inhibit SARS-CoV-2 replication and transcription (Qu et al., 2021).

Dihydroergotamine is a mixture of semi-synthetic ergot alkaloids mainly used for age-related cognitive impairment. This drug is used for the elderly with degenerative brain circulation disorders, senile dementia, cerebral arteriosclerosis, and stroke sequelae caused by dizziness, headache, attention loss, memory loss, depression, and fatigue (Bicalho et al., 2008). Dihydroergotamine formulations have improved efficacy, convenience, and tolerability in the treatment of migraine. It is also available as an intravenous, intramuscular, subcutaneous, or nasal spray (Chang et al., 2022; Johnson and Freitag, 2022; VanderPluym et al., 2022)

### Binding free energy calculated by MM/GBSA

The binding free energy for the interface of 40S and Nsp1 of eight compounds was calculated. We calculated the binding free energy of five drugs by MM/GBSA through the simulation trajectory of 100 ns molecular dynamics simulations methods. Golvatinib has the strongest binding free energy, suggesting it can be tested their anti-SARS-CoV-2 infection *in vitro* (**Table 2**). Further studies are needed to determine the utility of this finding by prospective clinical trials and identify other meaningful drug combinations.

**Table 2:** The calculated binding energies of ligand to 40S-SARS-CoV-2-Nsp1 (BQ.1).

Energy^*^	Golvatinib	Gliquidone	Dihydroergotamine	Zosuquidar	Hypericin	Ergotamine	Cepharanthine	Dihydroergotoxine
ΔEvdw	-49.4391±2.5697	-33.4418±3.9743	-43.2312±2.8943	-39.9047±2.4225	-25.3739±3.0561	-31.9218±4.8499	-32.6064±2.6807	-35.6549±2.2857
ΔEele	-24.3505±5.4941	-76.7045±12.0552	-62.2767±6.9128	-19.0902±5.4125	-59.0825±7.3229	-77.7844±6.2286	-9.8976±3.9818	1.8989±9.3275
ΔGgb	45.9147±5.1201	81.6445±9.162	78.963±7.2207	33.9326±4.504	64.7853±4.7867	92.553±6.182	26.1038±4.0384	20.1497±7.8705
ΔGnp	-6.3254±0.3489	-5.0916±0.3879	-4.8695±0.2628	-4.9831±0.367	-3.9425±0.1307	-4.059±0.4499	-3.8151±0.3023	-4.5116±0.276
ΔGcal	-34.2002±2.2896	-33.5934±5.0024	-31.4144±2.9617	-30.0455±2.2643	-23.6135±1.9712	-21.2121±3.4431	-20.2153±2.876	-18.1179±2.2313

^*^ ΔE_vdw_ , van der Waals energy terms; ΔE_ele_ , electrostatic energy; ΔG_gb_, polar solvation free energy; ΔGnp, nonpolar solvation free energy; ΔGcal, final estimated binding free energy calculated from the above terms (kCal/mol). The numbers after the “±” sign are Standard deviations.

## Conclusion

Although the pathogenicity of Omicron decreases, it will still cause death and the risk of sequelae in high-risk groups. Therefore, we still need to continuously monitor the changes of Omicron and possible variants in terms of pathogenicity, severe rate, and risk of sequelae. Although therapies and vaccines have been developed to promote immune protection against infection, several SARS-CoV-2 variants can evade current monoclonal antibody therapies (Wibmer et al., 2021). This variation can partially evade the mRNA vaccine-induced protection (Rogosnitzky et al., 2021). We are also facing declining immunity against vaccines. Therefore, therapeutic interventions outside of the immune system are needed to curb the threat of COVID-19. Since these drugs have been used in the clinic, their safety and side effects are well studied. Therefore, using these drugs in clinical trials or in combination may improve the possibility of substituting the current effective COVID-19 treatments. In particular, the two drugs with the highest binding free energy, Golvatinib, and Gliquidone, can be considered candidates for the treatment of COVID-19. Moreover, these two drugs also have clear safety and efficacy clinical data used in elderly patients, which can be used as target drugs for middle-aged and elderly patients with Omicron infection.

## Data availability statement

The original contributions presented in the study are included in the article/supplementary material. Further inquiries can be directed to the corresponding author.

## Author contributions

MS and SD designed the study. MS performed most studies, but a few were carried out by GL, TD and PD. All authors contributed thoughts and advice. All authors contributed to the article and approved the submitted version.
